# Emerging Rapid Resistance Testing Methods for Clinical Microbiology Laboratories and Their Potential Impact on Patient Management

**DOI:** 10.1155/2014/375681

**Published:** 2014-09-17

**Authors:** Hagen Frickmann, Wycliffe Omurwa Masanta, Andreas E. Zautner

**Affiliations:** ^1^Fachbereich Tropenmedizin am Bernhard-Nocht-Institut, Bundeswehrkrankenhaus Hamburg, 20359 Hamburg, Germany; ^2^Institut für Medizinische Mikrobiologie, Virologie und Hygiene, Universitätsmedizin Rostock, 18057 Rostock, Germany; ^3^UMG-Labor, Abteilung Klinische Chemie/Zentrallabor, Universitätsmedizin Göttingen, 37075 Göttingen, Germany; ^4^Institut für Medizinische Mikrobiologie, Universitätsmedizin Göttingen, Kreuzbergring 57, 37075 Göttingen, Germany

## Abstract

Atypical and multidrug resistance, especially ESBL and carbapenemase expressing Enterobacteriaceae, is globally spreading. Therefore, it becomes increasingly difficult to achieve therapeutic success by calculated antibiotic therapy. Consequently, rapid antibiotic resistance testing is essential. Various molecular and mass spectrometry-based approaches have been introduced in diagnostic microbiology to speed up the providing of reliable resistance data. PCR- and sequencing-based approaches are the most expensive but the most frequently applied modes of testing, suitable for the detection of resistance genes even from primary material. Next generation sequencing, based either on assessment of allelic single nucleotide polymorphisms or on the detection of nonubiquitous resistance mechanisms might allow for sequence-based bacterial resistance testing comparable to viral resistance testing on the long term. Fluorescence *in situ* hybridization (FISH), based on specific binding of fluorescence-labeled oligonucleotide probes, provides a less expensive molecular bridging technique. It is particularly useful for detection of resistance mechanisms based on mutations in ribosomal RNA. Approaches based on MALDI-TOF-MS, alone or in combination with molecular techniques, like PCR/electrospray ionization MS or minisequencing provide the fastest resistance results from pure colonies or even primary samples with a growing number of protocols. This review details the various approaches of rapid resistance testing, their pros and cons, and their potential use for the diagnostic laboratory.

## 1. Introduction

Generation of antimicrobial susceptibility patterns remains one of the most important tasks of clinical microbiology laboratories. The effective calculated antimicrobial therapy of infectious disease patients is consistently challenged by the rapidly rising prevalence of resistant and multidrug- or even pandrug-resistant pathogens worldwide. In recent years, this trend was accompanied by a shift from Gram-positive to Gram-negative bacteria like multidrug-resistant Enterobacteriaceae strains (MRE; resistant to three or more classes of antibiotics) as well as multidrug-resistant nonfermenters (*Pseudomonas aeruginosa* and* Acinetobacter baumannii*) [1–4]. In particular, carbapenemase expressing Enterobacteriaceae coresistant to non-beta-lactam antibiotics like quinolones, aminoglycosides, colistin, and fosfomycin are a recent major public health concern [5–8]. Colonization by MRE is highly region and patient group specific. For example, in the French capital Paris, a tenfold increase in the intestinal colonization rate of healthy individuals with extended-spectrum beta-lactamase- (ESBL-) producing bacteria was observed during the last half decade [9]. ESBL colonization was with 4.6%, particularly in French children aged 6–24 months, significantly above average [10]. Long lasting persistence of MRE, as demonstrated by a Swedish and a French study, contributes to the increase in the MRE prevalence, sometimes even years after infection [11, 12]. A median MRE-colonization period of 12.5 months could be detected in a cohort of newborn children in Norway [13]. An English study was able to verify persistence of resistance genes even in the absence of antibiotic pressure [14]. Furthermore, the colonization rate also differs between healthy subjects and patients at risk. It could be demonstrated in a Korean endemic area that 20.3% of healthy individuals were colonized with ESBL producers, while high-risk patients were colonized in 42.5% of cases [15]. However, the risk of faecal colonization depends mainly on the local prevalence. For example, an ESBL prevalence of 65.7% has been demonstrated in healthy adults in Thailand [16], while another study showed an ESBL prevalence of 11.3% in outpatients in England [17]. Farm animals are another reservoir for multidrug-resistant bacteria. A survey in the Netherlands demonstrated that chickens are colonized with ESBL-producing Enterobacteriaceae to more than 70%, while swine and cattle are known reservoirs for livestock-associated methicillin resistant* Staphylococcus aureus* (laMRSA) [18].

The multiresistances of the Gram-negative bacteria represent a major challenge for the traditional culture-based microbiology. Furthermore, the limited treatment options for a calculated therapy and therewith the risk of an inappropriate therapy are an intensifying factor of this problem [19]. As a consequence morbidity and mortality of outpatient and nosocomial-acquired infections with multidrug-resistant Gram-negative bacteria are significantly increased. Similarly,* Mycobacterium tuberculosis* has posed a serious health threat as a result of multidrug resistance. In its 2013 global report on tuberculosis, WHO estimates that 3.6% (95% confidence interval: 2.1–5.2%) of new cases and 20.2% (95% confidence interval: 13.3–27.2%) of previously treated cases had multidrug-resistant (MDR) tuberculosis (defined as tuberculosis caused by* M. tuberculosis* isolates that are resistant to rifampicin and isoniazid) and 1.3 million TB deaths [20]. On the other hand, CMV resistance has been reported to be on the rise in transplant recipients [21, 22].

Information on antimicrobial susceptibility aids a clinician in prescribing an appropriate antimicrobial drug for a particular infection. Due to the rapid rise in antimicrobial resistance worldwide [1], it is becoming increasingly important for a clinician to rapidly receive information on the antimicrobial susceptibility profile of the isolated pathogen for appropriate treatment to be initiated. Traditionally, clinical microbiology laboratories have relied on phenotypic methods to determine the antibiotic susceptibility profiles of pathogens [23]. These methods remain useful and have advantages such as low costs as well as being easy to perform and having established interpretation criteria. But they lack the ability to generate timely susceptibility results, hence delaying initiation of treatment [24]. Furthermore, currently there is a need to establish adequate and standardized screening and isolation procedures for carbapenemase-producing bacteria especially in risk patients as well as in patients in which MRE colonization/infection has been previously shown. These limitations have been found to have consequences in patient management; for example, delay in the initiation of antibacterial treatment has led to increases in mortality [25] as well as in hospitalization time [26] and make it challenging to implement the back-end approach of the antimicrobial stewardship program, which has shown rewarding results in patient management and the fight against antimicrobial resistance [27].

In response to the limitations of phenotypic methods and the desires to improve patient management and curb the spread of antimicrobial resistance, rapid antimicrobial susceptibility testing methods are continuously developed. These methods have been found to identify a pathogen and its antimicrobial susceptibility profile within a short period of time. There are basically five different ways to accelerate susceptibility testing in clinical diagnostics: (I) bypassing conventional culture by direct detection of the pathogen or resistance mechanism in the primary sample; (II) bypassing plate or broth culture dependent susceptibility testing (secondary culture); (III) avoiding time consuming work steps/methods; (IV) increasing the sensitivity to the detection of the infectious agent; that means detecting the infectious agent in earlier disease stages at lower viral or microbial loads; and (V) earlier detection of an evolving drug resistance during treatment in spreading less susceptible quasispecies.

For example, real-time quantitative PCR (qPCR) has made it possible to detect multidrug-resistant tuberculosis (MDR TB) in a sample within an hour, hence immediately initiating appropriate treatment and control measures [28]. Also, MALDI-TOF mass spectrometry (MS) has made it possible to detect the most pathogens in a sample within minutes with high sensitivity and specificity [29]. In addition, these methods have made it possible to control the spread of resistant strains, reduce the length of patient stay in hospitals, and enhance the implementation of antimicrobial stewardship programs.

In this review, we detail the rapid antimicrobial susceptibility testing methods that have been developed recently. They include classical agglutination assays; molecular testing methods, for example, qPCR, DNA microarrays, Luminex xMAP assays, and next generation sequencing; fluorescence* in situ* hybridization (FISH); and mass spectrometry-based methods, for example, phyloproteomics, assays using stable isotope labeling of amino acids, mass spectrometric beta-lactamase assays, PCR/electrospray ionization-mass spectrometry (PCR/ESI MS), minisequencing, and mass spectrometry-based comparative sequence analysis (MSCSA). In addition, we discuss the impact that these techniques are likely to bring for the patient management and the reduction of antimicrobial resistance.

## 2. Agglutination Assays as Rapid Culture-Associated Options

Agglutination assays are based on a suspension of microparticles coated with specific antibodies, leading to agglutination in contact with their specific antigens. Such procedures are useful for a preliminary resistance screening from pure bacterial colonies if the resistance mechanism of interest is associated with a single antigen only, which is expressed on the surface of the pathogen. Accordingly, agglutination assays are unfeasible for the screening for complex resistance patterns, which may be associated with multiple structurally different families of enzymes as in the case of extended-spectrum beta-lactamases (ESBL) or carbapenemases in Gram-negative rod-shaped bacteria.

Agglutination assays for the rapid identification of bacterial resistance patterns are widely restricted to the identification of the penicillin binding protein 2a (PBP-2a), the major resistance determinant of Methicillin resistant* Staphylococcus aureus* (MRSA). Different agglutination kits show specificities of 91.3% to 100% if applied to MRSA colony material [30–32]. The sensitivity is even more restricted, ranging between 82.7% and 94.1% [30–32]. If sufficient quantities of colony material are used, agglutination testing allows for the identification of small-colony variant MRSA strains as well [33].

The lack of sensitivity seems to be associated with certain staphylococcal cassette chromosome (*SCC-mecA*) types with type IV scoring particularly poor [31]. Furthermore, agglutination kits are only positive if methicillin resistance is due to the* mecA* gene. If* mecC*, a divergent* mecA* homologue, is the cause of the resistance, agglutination usually fails as observed for 10 out of 10* mecC*-positive, live-stock associated MRSA strains [34].

Of note, agglutination based PBP-2a testing is possible from liquid sample materials as well. However, the sensitivity is poor. From blood culture pellets, PBP-2a agglutination showed sensitivity of only 18% in a recent study. In contrast, specificity was excellent with 100% [35].

## 3. Genotypic Antimicrobial Resistant Detection Methods

The usage of genotypic methods in the rapid detection of antimicrobial resistance genes is gradually shifting from academic research laboratories to diagnostic laboratories and point-of-care testing. The attractiveness of these methods in determination of antimicrobial resistance has been attributed to two factors: firstly, their capability to generate results within a short time as compared to phenotypic methods; secondly, their capability to detect antimicrobial genes directly from the patient sample without necessarily waiting for culture results [36]. These two attributes aid clinicians in prescribing appropriate treatment to patients at the opportune time, hence making a positive contribution to antimicrobial stewardship programs [27]. However, genotypic tools for the detection of antimicrobial resistance may generate false negative results due to (i) their inability to detect new resistance mechanisms or (ii) false-positive results, because they may detect inactive or incomplete resistance genes in a specimen, which have not inferred resistance to the antimicrobial drug under test [37].

Current genotypic methods that are used for the rapid detection of antimicrobial resistance genes include (i) nucleic acid amplification methods, particularly real-time quantitative PCR (qPCR); (ii) DNA hybridization based methods, particularly DNA microarrays; (iii) Luminex xMAP technology; and (iv) next generation sequencing methods. Below is a brief description on the application of each of these molecular methods for the rapid detection of antimicrobial resistance.

### 3.1. Nucleic Acid Amplification Methods

Recently, one of the PCR techniques that has received a wide application in clinical microbiology is the quantitative real-time PCR (qPCR) technique [38]. This has been attributed to its flexibility and capability to rapidly and simultaneously identify multiple pathogens in a clinical specimen and the presence of antimicrobial resistance genes in the identified pathogens [39]. As a result, numerous qPCR assays for rapid identification of pathogens in clinical specimens have been developed but most of the available qPCR assays for detection of microbial resistance genes are limited to the detection of antibiotic resistance. In short, most of the available commercial qPCR assays detect the presence of* mecA* and* mecC*, which confer methicillin resistance in* S. aureus*; the* vanA* and* vanB* genes, which confer glycopeptide resistance; and genes that encode extended-spectrum *β*-lactamases (for detailed review on each assay see Maurin, 2012 [39]). One outstanding feature of all these qPCR assays is their capability to simultaneously and accurately detect resistance genes within a remarkably shorter time period of 4–6 hours. Similarly, qPCR assays for rapid detection of resistance against rifampin (RIF) and isoniazid (INH) have been introduced. Ramirez and coworkers have recently combined qPCR and high-resolution melt (HRM) technology to develop an assay, which rapidly and simultaneously identifies multidrug-resistant* M. tuberculosis*, mutations in the* rpoB* gene conferring resistance to RIF, and mutations in the* katG* and* inhA* genes conferring resistance to INH [28]. This assay produces results within 6 hours as compared to GenoType MTBDRplus assay (Hain Lifescience GmbH, Germany) and culture susceptibility testing, which take 8 hours and 56 days to generate results. In the recent time, several in-house qPCR assays for rapid and simultaneous detection of genes encoding* Klebsiella pneumoniae* carbapenemase (bla_KPC_) and New Delhi metallo-*β*-lactamase (bla_NDM_) in Gram-negative rod-shaped bacteria [40–43] have been introduced. Similarly, several in-house qPCR assays for rapid and simultaneous detection of bla_OXA-48_, bla_VIM_, and bla_IMP_ carbapenemase genes in Enterobacteriaceae have been established [44–46].

PCR-based MRSA testing has found wide applications in microbiological routine laboratories. Next to in-house assays, commercially available molecular MRSA testing platforms comprise, for example, BD GeneOhm MRSA (Becton Dickinson, Heidelberg, Germany), GT MRSA Direct/GQ MRSA (Hain Lifescience, Nehren, Germany), Hyplex StaphyloResist (Amplex, Gießen, Germany), LightCycler (Roche Diagnostics Ltd., Rotkreuz, Switzerland) kits like LC MRSA Advanced, Cepheid Xpert/Gene Expert (Cepheid, Sunnyvale, CA, USA), and TIB Molbiol LightMix MRSA (TIB Molbiol, Berlin, Germany). All test systems showed reliable results in a recent external laboratory control evaluation in Germany [47]. Similarly, commercial PCR assays for the detection of ESBL-associated bla_CTX-M_ beta-lactamases and only partially ESBL-associated bla_TEM_ and bla_SHV_ as well as OXA1-type carbapenemases (the latter combined in a consensus run) were introduced (Amplex, Gießen, Germany) [48]. Similar multiplex PCR systems are available for the most frequently detected carbapenemases, which are particularly useful for the follow-up during hospital outbreak events (Amplex, Gießen, Germany), even from primary sample materials [49, 50]. The switch of molecular carbanemase detection to robust loop-mediated isothermal amplification (LAMP) [46] allows for commercial point-of-care testing (POCT) compatible test solutions for bedside testing, for example, the eazyplex SuperBug CRE system (Amplex, Gießen, Germany) which provides results within 10 minutes. However, the great number of different possible cephalosporin and carbapenem resistance mechanisms finally exceeds any multiplexing capacity if completeness is aspired.

Nevertheless, in addition to rapid and simultaneous providing of reliable results, qPCR has been found to be affordable, sensitive, specific, user friendly, not space demanding, and deliverable [37–39, 51]. Due to these attributes, qPCR has found various applications in point-of-care testing (POCT). For example, the Xpert MTB/RIF test (Cepheid, Sunnyvale, CA, USA) is a qPCR-based assay that has been developed to rapidly and simultaneously detect* M. tuberculosis* and rifampicin (USAN: rifampin) resistance. To evaluate its usefulness in POCT, a large multicentre study involving 6069 cases from six unrelated sites was performed. In this study, Xpert MTB/RIF detected rifampicin resistance cases in 1 hour as compared to line-probe assay and phenotypic drug susceptibility testing that detected the same cases in 20 days and 106 days, respectively [52]. As mentioned above, a similar GenXpert-based POCT test for MRSA screening from clinical sample materials is available as well.

Multiplex PCR assays have also been developed to rapidly and simultaneously identify multiple pathogens in clinical specimens as well as the presence of antimicrobial resistance genes in the identified pathogens. Strommenger and coworkers developed a multiplex PCR, which simultaneously detects 9 resistance genes in* S. aureus* directly from clinical specimen within 6 hours [53]. These 9 resistance genes include* mecA* (methicillin resistance),* aacA*-*aphD* (aminoglycoside resistance),* tetK*,* tetM* (tetracycline resistance),* ermA*,* ermC* (macrolide-lincosamide-streptogramin B resistance),* vatA*,* vatB*, and* vatC* (streptogramin A resistance) [53].

Like qPCR, multiplex PCR assays were used as POC tests to facilitate patient management. One example is the multiplex PCR-based Unyvero Pneumonia Application (UPA) assay (Curetis AG, Holzgerlingen, Germany) that has been developed to rapidly and simultaneously detect 18 bacterial species,* Pneumocystis jirovecii* and 22 resistance markers from respiratory specimens (http://www.curetis.com/). In one of the studies showing its suitability for POC testing, the UPA assay detected multiple antibiotic resistances within 1 hour (as compared to phenotypic methods that took 96 hours) in a group of 56 hospitalized patients with respiratory tract infections who were under treatment. This finding influenced the modification of treatment in fifteen patients with severe pneumonia leading to their recovery [54]. The UPA assay is, of course, not able to replace conventional testing due to its design because it is not able to detect further microbial species and resistance mechanisms besides the implemented ones.

The continuous development of PCR-based assays with the capability to rapidly and simultaneously detect pathogens and presence of resistance genes in specimens coupled with their application in POCT may further improve the management of patients as long as appropriate quality control is ensured.

### 3.2. DNA Microarray Technology

The biggest challenge associated with the unprecedented rise of antimicrobial drug resistance worldwide is the scarce availability of assays that are able to rapidly and simultaneously identify a causative pathogen and generate its antimicrobial resistance profile. Recent oligonucleotide-based DNA microarrays match this challenge. In a recent study, Zhang and coworkers described that CapitalBio DNA microarray (CapitalBio Corp.) could in a mean time of 5.8 hours simultaneously identify* Mycobacterium* species and detect mutations that confer isoniazid (INH) and rifampicin (RMP) resistance in specimens collected from spinal tuberculosis patients as compared to conventional culture and drug susceptibility testing which took a mean time of 56.8 days [55]. Briefly, oligonucleotide probes, which had been designed to identify* Mycobacterium* species based on 16S rRNA sequences and mutations of* rpoB*,* inhA*, and* katG* that confer INH and RMP resistance, were covalently linked to the surface of aldehyde-activated slides. DNA was extracted from specimens. PCR was used to amplify the resistance genes and amplicons hybridized on the slides. The emitted fluorescent signals were analyzed. Guo and coworkers evaluated the ability of a biochip, which is based on the same principle to rapidly and simultaneously identify multidrug-resistant* M. tuberculosis* (MRTB) and mutations of* rpoB*,* inhA,* and* katG* that confer INH and RMP resistance in clinical sputum specimens [56]. This group found that the biochip could in a mean time of 6 hours simultaneously identify* M. tuberculosis* and detect mutations that confer INH and RMP resistance.

Recent reports have also reported the availability of Check-Point's ESBL/KPC DNA microarray for the identification and detection of extended-spectrum *β*-lactamases (ESBLs) and* Klebsiella pneumoniae* carbapenemases (KPC carbapenemases) [57, 58]. This array uses a methodology known as multiplex ligation detection to identify ESBL-associated or at least partially ESBL-associated genes (bla_TEM_, bla_SHV_, and bla_CTX-M_) and bla_KPC_ genes (for details see [57]). In a study to evaluate the rapidness at which this array could identify and detect these genes, Naas and coworkers found that Check-Point's ESBL/KPC DNA microarray could identify them in 7-8 hours as compared to conventional susceptibility testing that took a mean time of 54 hours. Similar results were observed by Willemsen and coworkers in a study that was aimed at evaluating the rapidness at which this array could identify and detect these ESBL/KPC genes in hospitals in the Netherlands [58]. In addition to detecting and identifying ESBL/KPC resistance in gastrointestinal tract infections caused by Enterobacteriaceae, Check-Point's ESBL/KPC DNA microarray has also been used to detect and identify KPC resistance in hospital-acquired pneumonia caused by* Klebsiella pneumoniae* [59]. Based on these experiences, the Check-MDR CT 102 DNA microarray for the detection of the most prevalent carbapenemase genes (bla_NDM_, bla_VIM_, bla_KPC_, bla_OXA-48_, and bla_IMP_) and extended-spectrum *β*-lactamase- (ESBL-) related gene families (bla_SHV_, bla_TEM_, and bla_CTX-M_) has been developed. The evaluation of the rapidness of the Check-MDR CT 102 DNA microarray to detect these genes has shown that it yields results 5 hours faster than Check-Point's ESBL/KPC DNA microarray [60].

At present, the DNA microarray technology is mostly used in the routine detection of antimicrobial resistance of TB and HIV [61–66]. The routine use of systems such as MVPlex (Genaco Biomedical Products, Huntsville, USA) and StaphPlex systems (Genaco Biomedical Products, Huntsville, USA), which combine both qPCR and DNA microarray technology, suggest that independent DNA microarray technology might find further applications in the routine clinical microbiology [67, 68]. The MVPlex system detects the* nuc*,* mecA*, (SCCmec)-*orfX*,* vanA*,* vanB*,* ddl*, and* tuf* genes to screen for MRSA in nasal swabs [69], and the related StaphPlex system performs simultaneous species-level identification (*nuc* versus* tuf*) and detection of* mecA*,* aacA, ermA*,* ermC*,* tetM*, and* tetK* as well as Panton-Valentine leukocidin (PVL) for the rapid detection and characterization of staphylococci directly from positive blood culture bottles [70].

### 3.3. Luminex xMAP Technology

The description of cooccurring single nucleotide polymorphism (SNP) mutations in antimicrobial resistance associated genes allows for targeted resistance testing. For example, unequivocally genetic studies have proven that there are 5 different mutations in quinolone resistance-determining region (QRDR) of* gyrA*,* gyrB,* and* parE* within* Salmonella typhi* [71]. Similar studies have also shown distinct mutations in the quinolone resistance-determining region (QRDR) of* gyrA* within* Campylobacter jejuni* and* Campylobacter coli* [72].

Rapid simultaneous detection of cooccurring single nucleotide polymorphism (SNP) mutations in antimicrobial resistance associated genes remains, however, challenging. Most molecular assays such as qPCR and pyrosequencing lack the capability to simultaneously detect cooccurring single nucleotide polymorphism (SNP) mutations in different genes in a given specimen [73]. However, this challenge has been overcome by Luminex xMAP Technology, a multiplexing technology, which allows for simultaneous detection of multiple nucleic acid sequences in a single reaction [74]. During operation, microtiter plates are loaded with microspheres, that is, coated and color-coded beads. The microspheres are mixed with purified nucleic acids of the test organism and allowed to hybridize, emitting monochromatic light, which the Luminex analyzer reads and interprets. At present, this technology has been used to simultaneously detect 11 mutations in* gyrA*,* gyrB,* and* parE* of* Salmonella* Typhi and* Salmonella *Paratyphi A [75]. Further, it has been used to simultaneously detect mutations in* gyrA* of* C. jejuni* and* C. coli* [76]. In comparison to sequencing and microarray technology, Luminex xMAP Technology has been found to be flexible, rapid, and cost effective [74–76].

### 3.4. Next Generation Sequencing (NGS)

Near whole genome sequencing (WGS) or next generation sequencing (NGS) allows for the assessment of bacterial genomes within several hours. A variety of different technological solutions have been introduced, including laser printer sized benchtop devices like 454 GS Junior (Roche, Basel, Switzerland), MiSeq (Illumina, San Diego, CA, USA), and Ion Torrent PGM (Life Technologies, Grand Island, NY, USA). In a previous analysis, the MiSeq (Illumina) system scored best regarding both throughput per run and error rates, while both the 454 GS Junior (Roche) and the Ion Torrent PGM (Life Technologies) systems were prone to homopolymer-associated indel errors [77].

Result interpretation of whole bacterial genomes is based on either allelic comparisons [78] or single nucleotide polymorphism (SNP) analysis [79]. Data assessment and interpretation can be facilitated by commercial software packages like SeqSphere+ (Ridom BIOINFORMATICS Ltd., Münster, Germany) or BioNumerics (Applied Maths, Sint-Martens-Latem, Belgium).

NGS allows for resistance identification by the presence of the underlying mechanism rather than just in pharmacodynamic terms [80], so it may revolutionize microbial resistance testing on the long term. This comprises the identification and characterization of resistance genes encoding for extended-spectrum *β*-lactamases (e.g., *bla*_CTX-M_, *bla*_TEM_, and *bla*_SHV_), plasmid-mediated AmpCs (e.g., *bla*_CMY_), quinolone resistance (e.g., mutations in* gyrA*,* parC*, or* qnr* elements), aminoglycoside resistance (e.g., aminoglycosides modifying enzymes, 16S rRNA methylases), or carbapenemases (e.g., *bla*_KPC_, *bla*_NDM_) [81].

NGS-based resistance testing is of particular interest for slowly growing infectious agents with atypical resistance patterns like multidrug-resistant (MDR) or extensive-drug resistance (XDR)* M. tuberculosis*, for which rapid identification or exclusion of resistance determinants is of high relevance for the therapeutic approach. Ion Torrent full-gene sequencing with consecutive complete genetic analysis within 5 days (Table 5) allowed for reliable resistance detection in* M. tuberculosis* isolates of Burmese, Hmong, and Indian immigrants in the USA [82]. Similar WGS data were described for drug-resistant strains from Russia, harbouring almost all known drug-resistance associated mutations [83]. In a direct comparison of Ion Torrent sequencing with phenotypic Bactec MGIT 960 (Becton Dickinson, Franklin Lakes, NJ, USA) analysis and genotypic Hain line-probe assay (LPA) (Hain Lifescience Ltd., Nehren, Germany), there was complete concordance of NGS to phenotypic resistance and genotypic* rpoB* and* katG* results for the analyzed* M. tuberculosis* isolates. Even more, Ion Torrent sequencing detected uncommon substitutions and previously uncharacterized resistance mutations in* rpoB*,* rrs*, and* pncA* [84]. Further, NGS is able to discriminate mixed mycobacterial genotypes in patient isolates based on single nucleotide variations (SNVs) [85]. So it might be suitable to identify resistance mutations in genotypes that occur in minor proportions only.

However, NGS-based resistance testing is not restricted to mycobacteria. Recently, NGS was used to identify transmissible plasmids in multidrug-resistant* E. coli* isolates expressing an ESBL phenotype and transferring their cefotaxime resistance marker at high frequency in laboratory conjugation experiments [86]. High-throughput sequencing successfully proved to be a valuable tool for tracing resistance plasmids in the course of outbreaks as well [87]. However, a commercial NGS assay (Hospital Acquired Infection BioDetection System, Pathogenica, Boston, MA, USA) for investigations of outbreaks with ESBL-positive Enterobacteriaceae showed good sensitivity (98%) but failed to discriminate between ESBL and non-ESBL TEM and SHV beta-lactamases or to specify CTX-M genes by group [88].

Current obstacles to a routine use of NGS technologies in diagnostic microbiology and resistance testing comprise costs and scarcely available user-friendly bioinformatics platforms [89]. Nevertheless, NGS technologies provide high-resolution genotyping in a short time frame of only two to five days [89]. Therefore, NGS/WGS in the microbiological laboratory will be the logical next step for the routine diagnosis of infection and the prediction of antimicrobial susceptibility [90], potentially replacing traditional cultural approaches on the intermediate or long term.

## 4. Fluorescence *In Situ* Hybridization (FISH) for the Detection of Bacterial Resistance

FISH (fluorescence* in situ* hybridization) is a cheap and convenient option for the identification and resistance testing of bacterial pathogens. Traditional FISH is based on specific hybridization of short, usually 18–25 bases long, fluorescent-labelled, single-stranded oligonucleotide probes to ribosomal RNA (rRNA) of the target organism with subsequent analysis under the fluorescence microscope, usually allowing for the identification of microbes at genus or species level. In principle, each kind of intracellular RNA can be hybridized with FISH probes. However, rRNA is particularly well suited as a FISH target, because ribosomes are numerous in a protein-synthesizing cell, thus allowing for a boostering of fluorescence intensity [91].

This traditional FISH method is both rapid and easy to standardize, so it can be applied for molecular rapid testing. Small modifications of the procedure comprise the use of patent-protected, commercial peptide nucleic acid (PNA) probes or probes containing locked nucleic acids (LNA) instead of simple single-stranded DNA probes. PNA-FISH technology reduces nonspecific probe attachment due to the electrically neutral backbone of the oligonucleotides and is recommendable for routine diagnostics due to a higher degree of standardization. However, patent-protected PNA probes are expensive, although they are well suited for the diagnostic routine setting [92].

FISH is particularly suitable for the detection of resistance determinants if two prerequisites are guaranteed. Ribosomally mediated resistance, for example, affecting antibiotic drugs like macrolide or linezolid, is well suited, because ribosomal RNA copies are numerous in living cells, allowing for bright fluorescence signals. Further, FISH can be successfully applied if only one or few variable bases provide resistance, so there is no need for a large number of probes in the probe panel.

These prerequisites are fulfilled in case of clarithromycin resistance testing in* Helicobacter pylori*. Therefore, FISH-based resistance testing was early evaluated for this indication [93]. Clarithromycin in* H. pylori* is basically mediated by three point mutations in the ribosomal 23S rRNA [94] which can be addressed by three described FISH probes: ClaR1, ClaR2, and ClaR3 [93] (Table 1). While ClaR1 is associated with a minimum inhibitory concentration (MIC) of >64 mg/L, ClaR2 and ClaR3 are associated with varying MICs between 8 mg/L and 64 mg/L [94].

The FISH probes for clarithromycin resistance testing in* H. pylori* were successfully applied to bacteria both from culture and in bioptic material and extensively assessed in various studies [93, 95–97]. Reliable test results can even be achieved in formalin-fixed, paraffin-embedded tissue after adequate deparaffination [98]. The combined use of probes labelled with different fluorescence molecules allows for the identification of coinfections with clarithromycin-sensitive and -resistant* H. pylori* strains by FISH [99].

Commercial test providers distributed the robust and easy-to-apply procedure. In one study with such a commercial test kit [100], a sensitivity of 90% and a specificity of 100% were achieved for the detection of clarithromycin-resistant* H. pylori* within bioptic material. In another study, occasional false-positive* H. pylori* detections were generated [101], although the results of FISH-based resistance testing of correctly identified* H. pylori* proved to be reliable. Recently, a PNA probe-based approach for clarithromycin resistance testing in* H. pylori* showed perfect matching with PCR/sequencing in a retrospective study with formalin-fixed, paraffin-embedded tissues (Table 2) [102].

Similar to* H. pylori*, FISH-based clarithromycin resistance testing could be successfully demonstrated for thermotolerant* Campylobacter* spp. with a wild-type probe and a clarithromycin resistance probe targeting the A2059G mutation in the 23S rRNA gene (Table 3). The observed sensitivity and specificity with culture material were 100% [103].

Comparable to clarithromycin resistance, linezolid resistance is ribosomally mediated. In enterococci, it is typically caused by a 2567G>T base substitution in the 23S rRNA (Table 4). In a collection of 106 enterococcal isolates, a corresponding linezolid resistance FISH assay succeeded in predicting phenotypic resistance in 100% of cases [104]. Even a single mutated allele was associated with strong fluorescence signals.

First successful attempts of FISH-based resistance testing were described for non-rRNA-based resistance mechanisms as well. FISH-based detection of bla_SHV-238/240_, one of the genes coding for extended-spectrum *β*-lactamases (ESBL), is an example of a non-rRNA-based FISH protocol for detecting a particular resistance determinant using the probe 5′-GAC-CGG-AGC-TAG-CAA-GCG-3′ [105]. However, the ESBL phenotype can be associated with a variety of different alleles, so this particular probe will be of use only in case of a specific suspicion, for example, during an outbreak. Accordingly, such a procedure will be reserved for very few if any indications in the diagnostic routine.

Further progression of FISH technology comprises signal-amplified, catalyzed reported deposition (CARD) FISH; doubly labeled oligonucleotide probe- (DOPE-) based FISH; combinatorial labelling and spectral imaging (CLASI) FISH; and the combination of FISH with other diagnostic approaches as well as FISH procedures for gene identification, requiring* in situ* amplification of the respective gene as in case of the rolling circle amplification (RCA) FISH [106]. RCA-FISH was successfully applied for the identification of the* mecA* gene in Methicillin resistant* Staphylococcus aureus* (MRSA) based on the mecA-probes MR-1 5′-AAG-GAG-GAT-ATT-GAT-GAA-AAA-GA-3′ and MR-2 5′-GGA-AGA-AAA-ATA-TTA-TTT-CCA-AAG-AAA-A-3′ [107].

**Figure 1:**
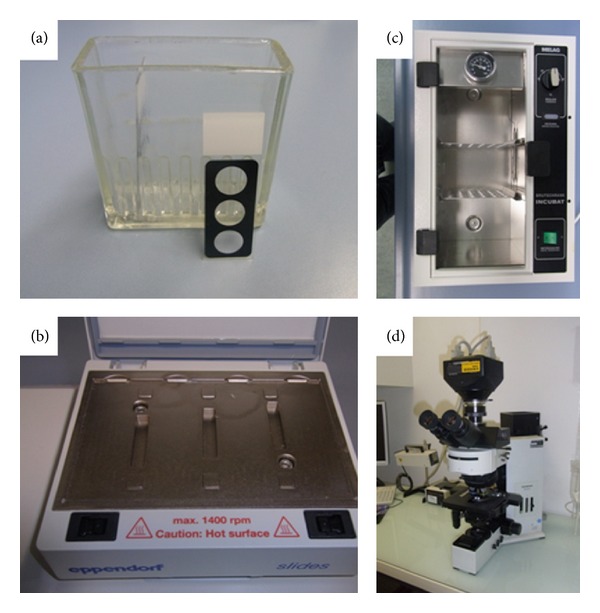
Little equipment—as here exemplified by material from the Institute for Microbiology, Virology and Hygiene, University Medical Center Rostock—is required for performing FISH analyses. (a) Glass apparatus for fixing and washing of slides. (b) Slide chamber, allowing for a rapid and steady heat transmission. (c) Incubator for the washing step. (d) Multichannel fluorescence microscope.

FISH-based detection of resistance determinants is a promising diagnostic approach due to its rapidity, convenience, and cost effectiveness. The associated rapid detection of antimicrobial resistance may lead to early resistance-adapted optimization of antimicrobial therapy with associated benefits for the patient's health. The main advantage of FISH is its potential use for resistance testing directly from primary material including tissue with low effort. So FISH can also be applied in resource-limited settings where expensive technologies are not available (Figure 1). In contrast to PCR, FISH can also attribute a particular resistance mechanism to a microscopically observed bacterium.

However, so far, FISH is restricted to very few indications for which protocols have been described. As a further drawback, standardization of FISH-based resistance testing is widely missing. If applied from primary sample materials like tissue, tissue autofluorescence has to be considered, requiring considerable experience to interpret such diagnostic results. To reduce potential interpretation errors, FISH from tissue further requires counterstaining with a pan-eubacterial FISH probe and nonspecific DNA staining, for example, with DAPI (4′,6-diamidino-2-phenylindole), to confirm the presence of nucleic acids of the detected pathogens as recently demanded [108].

Given all these limitations, FISH for resistance testing will presumably stay a bridging technology until amplification-based technologies will be available as easy-to-apply and cost-efficient benchtop systems on the market.

## 5. Direct Fluorescent Imaging of Resistance Determinants by Fluorescence Resonance Energy Transfer (FRET)

Nonnucleotide probes labelled with reporter and quencher molecules, allowing for fluorescence energy transfer (FRET), can be used to detect enzymatic resistance mechanisms as described for *β*-lactamases [109]. After enzymatic hydrolyzation of probes to separate the quencher from the reporter, the hydrolyzed probes attach the resistance enzymes as reactive electrophiles. However, this mechanism has so far been only described for *β*-lactamases in a proof-of-principle analysis [109] and broad evaluation studies are missing. Its practical relevance for the microbiological routine diagnostics will require further evaluation.

## 6. Mass Spectrometric Approaches

Matrix-assisted laser desorption ionization time-of-flight mass spectrometry- (MALDI-TOF MS-) based intact cell mass spectrometry (ICMS) has recently advanced to the standard method for species identification for cultured bacteria and fungi [24, 110–114]. Promising approaches have been made using ICMS spectra for subspecies identification [115]. This technique bears a high potential for the fast identification of susceptibility associated biomarker ions that is lately only marginally realized in clinical routine diagnostics. Thus, phyloproteomic approaches help to identify indirectly mostly chromosomal encoded resistance genes by identifying phylogenetic relatedness [116–121]. MS can be used to detect changes in the bacterial or fungal proteome induced by exposition to antimicrobials [24, 122–124]. Whole proteome changes in consequence of exposition to antimicrobials can be also detected using stable isotope labeled amino acids (SILAC) [125, 126]. One very promising approach is the so-called mass spectrometric beta-lactamase (MSBL) assay [127–131], which is based on the mass spectrometric detection of hydrolyzed beta-lactams. Finally there is the combination of genotypic and mass spectrometric methods: PCR amplicons can be characterized by PCR/electrospray ionization-mass spectrometry (PCR/ESI MS) [132], and minisequencing [133, 134] and mass spectrometry-based comparative sequence analysis [135, 136] can be used to detect susceptibility changes associated with point mutations.

### 6.1. Prediction of Broad Spectrum Resistant Clonal Groups by Phyloproteomics

MALDI-TOF MS-based intact cell mass spectrometry (ICMS) is potentially able to characterize strains at the subspecies level and could act as useful tool for taxonomy and epidemiology [137, 138]. For the discrimination of representative strains particular biomarker ions that were completely present or absent as well as shifts in biomarker masses in a particular subset of strains were considered. Using different mathematical algorithms, it was, for example, feasible to discriminate* Salmonella enterica* ssp.* enterica* serovar Typhi from other less virulent* Salmonella enterica* ssp.* enterica* serotypes [139], to distinguish* Campylobacter jejuni* MLST-ST22 and ST45 from other MLST sequence types [140] or to perform phyloproteomic analysis of* Rhodococcus erythropolis* [141],* Pseudomonas putida* [142], or* Neisseria menigitidis* [143].

The first approaches to associate MS fingerprints with susceptibility patterns were designed to differentiate methicillin susceptible* Staphylococcus aureus* (MSSA) from methicillin resistant* Staphylococcus aureus* (MRSA) [144–148]. These were mostly not standardized and hardly reproducible. But relatively good reproducibility was demonstrated for the discrimination of the five major MRSA clonal complexes CC5, CC8, CC22, CC30, and CC45 corresponding to the five major PFGE MRSA types regardless of their methicillin sensitivity [149, 150]. A study by Lu and coworkers identified a set of biomarkers that were able to distinguish between methicillin resistant and vancomycin-intermediate* S. aureus* (VISA) strains and vancomycin-susceptible* S. aureus* strains, as well as between SCCmec types IV and V isolates and SCCmec types I–III isolates [151]. Further studies demonstrated that isogenic* S. aureus* lacking or artificially harboring SCCmec could not be distinguished in a mass range from 2000 to 15000 *m*/*z* [152], whereas isogenic MRSA, which spontaneously reverted to MSSA, could be discriminated by MALDI-TOF MS [153].

One study from New Zealand showed that the discrimination of* vanB* positive vancomycin-resistant* Enterococcus faecium* (VRE) and vancomycin-susceptible* E. faecium *using ICMS fingerprinting is feasible [121], but these findings were not reproducible in other areas. Thus it was speculated that this was just reflecting the specific epidemiological situation in New Zealand [125].

Other studies on* Clostridium difficile* demonstrated a sufficient discriminatory power of MALDI-TOF MS spectra analysis to recognize the PCR ribotypes 001, 027, and 126/078 [116]. Phyloproteomic analysis is a sufficient tool to identify high-virulent or multidrug-resistant strains of particular bacterial species if their virulence or their resistance is associated with phylogenetic and therewith phyloproteomic relatedness. Thus it is an up-and-coming technique not only for epidemiological surveys but also for individual patient management.

Compared to Gram-positive bacteria, Gram-negative bacteria are particularly problematic because their resistance genes are often encoded on plasmids, which can be easily exchanged with other Gram-negative bacteria even across species boundaries [154]. But some of the extended beta-lactamase genes (ESBL) and carbapenemases are associated with particular bacterial clonal complexes.* Klebsiella pneumoniae* ST258 (expressing KPC carbapenemase) and* E. coli* ST131, ST69, ST405, and ST393 (expressing ESBL) [155] belong to these clonal complexes.

Similar phyloproteomic analysis has been successfully demonstrated to discriminate between different subsets of* E. coli* strains [156]. Coupling MALDI-TOF MS with multivariate data analysis allows for discriminating ESBL-expressing* E. coli* B2 ST131 and D (ST69, ST393, and ST405) from other* E. coli* strains [117, 118].

One likely problem in the calculated treatment of* Bacteroides fragilis* infections is the possibility that some strains express a high-potential metallo-*β*-lactamase encoded by the gene* cfiA* [157]. The microbial species* B. fragilis* is subdivided into two divisions (I and II) and usually only isolates of division II harbor* cfiA*. Recently, two independent studies identified a set of biomarkers or precisely shifts in biomarker masses that help to distinguish both divisions using MALDI-TOF MS coupled with a cluster algorithm [119, 120].

### 6.2. Detection of Whole Proteome Changes Induced by Echinocandins

Echinocandins, namely, anidulafungin, caspofungin, and micafungin, are the treatment of choice for invasive and systemic infections with* Candida* and* Aspergillus* species. They also comprise important reserve antimicrobial agents especially in the case of infections with azole-resistant strains, for example,* Aspergillus* species. Due to the increasing use of echinocandins in the treatment of fungal infections, the prevalence of echinocandin-resistant isolates caused by mutations in the* fks1-3* (hypersensitive for the immunosuppressant FK560) genes increases [158]. Thus, rapid identification of azole and echinocandin susceptibility are needful for a successful therapy of systemic mycoses.

In a pioneer study, the feasibility of MALDI-TOF MS-based testing to estimate fluconazole susceptibility of* Candida albicans* was shown by Marinach and coworkers [122]. During the test procedure,* Candida* cells were incubated for 24 hours in liquid medium containing different concentrations of fluconazole. After harvesting and acid extraction of the* Candida* cell pellets, the supernatants were spotted on a MALDI-TOF target plate and mass spectra were recorded. Comparable to the estimation of minimal inhibitory concentrations (MIC), the so-called minimal profile changing concentration (MPCC), the lowest concentration of fluconazole at which changes in the mass spectrum were recordable, was estimated by comparing the mass spectra of the particular suspensions of the fluconazole dilution series. Remarkably, MPCC differed only in one dilution step from the MIC and therewith it is a comparably sufficient parameter reflecting antimicrobial susceptibility [122].

de Carolis and coworkers adapted this procedure to test* C. albicans*,* Candida glabrata*,* Candida parapsilosis*,* Candida krusei*,* Aspergillus fumigatus*, and* Aspergillus flavus* for echinocandin MICs that are due to mutations in* fks1* and, in the case of* C. glabrata*, also in* fks2* [123]. Additionally, they accelerated the data analysis by applying composite correlation index (CCI) analysis. The CCI value was calculated in comparison to reference spectra of the two extreme concentrations [123].

This procedure was further optimized by Vella and coworkers [124]. They reduced the incubation period down to 3 hours by incubating the yeast cell suspension without as well as with two different echinocandin concentrations corresponding to intermediate and complete resistance [124].

### 6.3. Stable Isotope Labeling by Amino Acids in Cell Culture (SILAC)

The successful application of mass spectrometry (MS) in the detection of antimicrobial resistance has also opened a door for the entry of another quantitative proteomics approach known as SILAC into the era of rapid detection of antibiotic resistance. This approach is based on the principle that proteins are made up of amino acids. Hence, cells grown in media supplemented with amino acids incorporate these amino acids into their cellular proteome [125]. In addition, protein profiles of a metabolically active cell reveal its metabolic activities at a specific time. Already established SILAC antimicrobial detection protocols to detect antibiotic resistance involve the growth of three cultures of the test strain. The first culture is grown in medium with normal (light) essential amino acids, the second culture is grown in media supplemented with labeled (heavy) essential amino acids, and the third culture is grown in media supplemented with both labeled (heavy) essential amino acids and the analyzed antimicrobial drug. These three cultures are mixed, their proteomes are extracted and measured by MS, and the peaks are compared. The test strain is classified as susceptible if its protein peak profile is similar to that of the first culture. On the other hand, it is classified as resistant if its protein peak profile is similar to the second culture [159]. This approach has been successfully used to differentiate methicillin susceptible* S. aureus* (MSSA) and methicillin resistant* S. aureus* (MRSA) [160]. Also, it has been successfully used to test the susceptibility of* P. aeruginosa* to three antibiotics of different classes with different modes of action: meropenem (*β*-lactam antibiotic), tobramycin (aminoglycoside), and ciprofloxacin (fluoroquinolone) [126]. In both cases, the results were assessed after 2 to 4 hours and the results were comparable to those obtained from minimum inhibitory concentration (MIC) testing. In addition to these advantages, SILAC is easy and straightforward to perform. For this reason, very soon it may be used to detect antimicrobial resistance in antiviral, antifungal, and antiparasitic drugs.

### 6.4. Mass Spectrometric *β*-Lactamase Assay

In contrast to the aforementioned mass spectrometric assays, the mass spectrometric *β*-lactamase assay (MSBL) is not based on the analysis of the bacterial proteome. The MSBL is based on the direct mass spectrometric detection of *β*-lactamase metabolites [127–131]. The procedure is as follows. First bacteria are suspended in a buffered solution with and for reference without a *β*-lactam antibiotic. This suspension is incubated for 1 to 3 hours. After centrifugation, the supernatants are analyzed by MALDI-TOF MS. Specific peaks (mass shifts) for intact and hydrolyzed *β*-lactams indicate functional presence of *β*-lactamases. It was demonstrated that the MSBL delivers results within 2.5 hours for bacteria inactivating ampicillin, piperacillin, cefotaxime, ceftazidime, ertapenem, imipenem, and meropenem [131]. Thus, particularly NDM-1, VIM-1/2, KPC-1-3, OXA-48, OXA-162, and IMP carbapenemase expression by Enterobacteriaceae,* Acinetobacter baumannii,* and* Pseudomonas* spp. was detectable [128, 130].

With a total turn-around-time after positive primary bacterial culture of circa 4 hours, this method is significantly faster than culture-based susceptibility testing [127–131].

### 6.5. Mass Spectrometric Analysis of PCR Products: PCR/ESI MS

PCR/electrospray ionization-mass spectrometry (PCR/ESI MS) combines, nucleic acid amplification with mass spectrometric analysis of the amplicons, which are brought into a gas phase using electrospray ionization. The major advantage of this technique is its high multiplexing capacity that enables the parallel detection of a wide panel of resistance genes. It was demonstrated that PCR/ESI MS is able to accurately detect nine different KPC carbapenemases (bla_KPC-2-10_) [132] as well as the* gyrA* and* parC* point mutations, which are associated with quinolone resistance in* A. baumannii *[161].

Also because of its high multiplexing capacity, PCR/ESI MS is a suitable tool for simultaneous (sub)species identification and resistance gene detection, which is of particular importance for the treatment of mycobacterial infections. On the one hand, it is necessary to distinguish nontuberculosis mycobacteria (NTM) from* M. tuberculosis*; on the other hand, multidrug-resistant tuberculosis (MDR-TB) strains must be detected. PCR/ESI MS-based assays have been developed to facilitate NTM species identification and parallel detection of resistance genes associated with rifampicin, isoniazid, ethambutol, and fluoroquinolone resistance in TB and NTM [162]. Moreover, there are enormous time savings compared to traditional mycobacterial culture and resistance testing via the agar proportion method [162–164].

The high sensitivity of PCR/ESI MS in the detection of hard-to-culture or even nonculturable bacteria makes it a reliable method for the direct detection of pathogens in hardly acquirable samples like heart valves [165] as well as for surveillance studies [166, 167].

### 6.6. Minisequencing-Primer Extension Followed by Matrix-Assisted Laser Desorption/Ionization Time-of-Flight Analysis (PEX/MALDI-TOF)

Another method that was also adapted for the rapid detection of ganciclovir resistance in HCMV (human cytomegalovirus) by Zürcher and coworkers is single nucleotide primer extension (also known as minisequencing or PinPoint assay) followed by matrix-assisted laser desorption/ionization time-of-flight analysis (PEX/MALDI-TOF) [134]. In general, the combination of PEX and MALDI-TOF MS is a cost-efficient high-throughput method for the detection of single nucleotide polymorphisms (SNPs) [133]. The PEX/MALDI-TOF workflow using patient plasma is as follows [134].

For the primer extension reaction, the reverse PEX primer (5′-CTT-GCC-GTT-CTC-CAA-C-3′) was added in high concentration. The 3′-end of the primer is located directly at the site of mutation (A594V; GCG/wild type → GTG/mutant) to be detected. The extension reaction catalyzed by a DNA polymerase is terminated in the case of a wild-type allele just after one nucleotide complementary to the mutated nucleotide and in the case of a mutant after two nucleotides by a didesoxynucleotide (ddNTP). Because of the molecular weight difference in consequence of the varying mass increase of the PEX primer, mutant and wild type can be discriminated using MALDI-TOF MS [133].

According to current standards, HCMV resistance testing is performed using Sanger sequencing [168]. By monitoring a patient cohort of five individuals using Sanger sequencing and PEX/MALDI-TOF, Zürcher et al. could demonstrate that the PEX/MALDI-TOF method is much more sensitive than the Sanger method. PEX/MALDI-TOF requires the presence of only 20%–30% of the ganciclovir unsusceptible HCMV quasispecies to reliably detect the resistance mutation [134]. In consequence, this method was able to detect the appearance of the UL97 resistance mutation already ten days after the “last wild-type only constitution,” whereas Sanger sequencing detected the appearance of the resistant subpopulation at day 20 [134]. Consequently, a ganciclovir therapy can be monitored by PEX/MALDI-TOF more contemporary. A necessary change in therapy may be done earlier, and critical time for the preservation of the graft and the patient can be saved.

A comparable test setup was designed to detect TEM-type ESBL in Enterobacteriaceae [169]. Conversion of TEM penicillinases to TEM-type ESBL is mostly due to amino acid substitutions at Ambler's positions: Glu104, Arg164, and Gly238 [170]. To detect these SNPs in the *bla*_TEM_ genes, a set of seven internal primers have been designed to bind near the three codons of Ambler's positions in such a way that the masses of all possible reactions products are maximally distant from each other and are easy to distinguish in the mass spectrum. All primers are used in one multiplex reaction. Thus it is feasible to detect different types of TEM-type ESBL in one reaction [169].

Other minisequencing protocols have been established to detect fluoroquinolone resistance related SNPs in* N. gonorrhoeae* [171], clarithromycin resistance in* Helicobacter pylori* [172], and rifampin and isoniazid-resistance in* M. tuberculosis* [173].

### 6.7. MSCSA-Mass Spectrometry-Based Comparative Sequence Analysis to Detect Ganciclovir Resistance

Mass spectrometry-based comparative sequence analysis (MSCSA) was initially established by Honisch and coworkers (SEQUENOM, San Diego, USA) for the genotyping of bacteria using mass spectrometric fingerprinting of the standard multilocus sequence typing (MLST) loci [135].

The MSCSA principle was adapted to facilitate the detection of mutations in the UL97 gene to detect ganciclovir resistance of human cytomegalovirus (HCMV) [136].

HCMV reactivation occurs frequently in consequence of immune suppression especially after stem cell and solid organ transplantation [174]. Thus, HCMV infection may lead to graft dysfunction or even rejection. To counteract this, antiviral treatment with the analogue of 2′-deoxy-guanosine ganciclovir is indicated [175]. Under therapy, which may span several months, it is necessary to monitor the emergence of resistance and possibly switch to other drugs such as the more toxic foscarnet [176]. Ganciclovir resistance is typically a consequence of single nucleotide polymorphisms in the 3′-region of the UL97 kinase gene encoding a viral kinase, which activates ganciclovir by phosphorylation [177].

These UL97 single nucleotide polymorphisms are detected by MSCSA as follows: after DNA isolation from EDTA-plasma samples, the 3′-region of the UL97 is amplified in two amplicons using T7-promotor-tagged forward primers and SP6-tagged reverse primers. Both amplicons are* in vitro* transcribed in two separate reactions using T7 and SP6 RNA polymerase followed by cytosine or uracil specific RNaseA cleavage of plus and minus strand RNA transcripts. After this, all four obtained RNaseA cleavage products are transferred to a SpectroCHIP array (SEQUENOM, San Diego, USA). MALDI-TOF mass spectra are recorded and* in silico* compared to calculated MS spectra of reference sequences. Based on the obtained data, the UL97 sequence can be assembled and thereby the presence of a ganciclovir resistance associated single nucleotide polymorphism can be detected [136]. Due to the automation of post-PCR processing and analysis as well as reduced hands-on time, acceleration of the detection process of ganciclovir resistance can be achieved.

## 7. Conclusions and Outlook

To solve the increasing problem of a worldwide rising prevalence of infections due to multidrug- or even pan-drug-resistant bacteria, medical microbiology has to establish a new generation of rapid resistance testing assays. The key features of these new assays should be significant reduction of turn-around-time (Table 5) and a high multiplexing capacity, because of the already mentioned shift from Gram-positive to Gram-negative multidrug-resistant bacteria in recent years with various resistance mechanisms [1–4]. So, MRSA detection simply means detection of the penicillin binding protein 2A (PBP2A), the SCCmec genetic element, respectively [178]. Detection of vancomycin-resistant* S. aureus* (VRSA) as well as vancomycin-resistant enterococci (VRE) means the detection of Van-A, Van-B, and rarely Van-C [179].

In contrast to this situation in Gram-positive bacteria, multidrug resistance in Gram-negative bacteria is due to the expression of extended-spectrum *β*-lactamases (ESBLs), carbapenemases, aminoglycoside-blocking 16S rRNA methylases, and many other mechanisms associated with several hundreds of gene variants/mutations [4–8]. The more these resistance genes can be detected in parallel, the higher the probability of an exact determination of a particular susceptibility pattern is.

But rapid resistance testing is only one key to the solution of this problem, especially because the multiplexing capacities of the individual assays are limited and the costs are too high. Thus, resistance surveillance programs are and have been established at different levels: hospital-wide, regional, and international. For example, some hospitals introduced a general ESBL screening in analogy to the MRSA screening in high-risk groups. In recent years, various studies were carried out to identify the ESBL-transmission rate in maximum care hospitals and in households with ESBL-colonized individuals. The studies showed that the ESBL-transmission rate of 1.5% to 4.5% is relatively low if compliance with standard hygiene measures is guaranteed [180, 181]. In contrast, the ESBL-transmission rate in households with common food preparation was 25% and therewith comparable high as the MRSA-transmission rate [181, 182]. A prospective study demonstrated a relatively high prevalence of 15% for ESBL-producing Enterobacteriaceae on admission, but these strains were involved in only 10% of the infections at admission time [183]. Such regional surveillance studies form the basis for national and international surveillance statistics such as those published by the European Antimicrobial Resistance Surveillance Network (EARS-Net). Such surveillance studies on the prevalence of certain ESBL and carbapenemase subtypes can contribute to the identification of resistance mechanisms of the quantitatively biggest importance, which should be included in Gram-negative test panels. Thus, appropriate surveillance studies contribute to the solution of the problem of limited multiplexing capacity at least partially.

As recently predicted, next generation sequencing (NGS) with its high multiplexing capacity will soon be part of routine diagnostics, more and more replacing cultural approaches as an accurate and cheap procedure in routine clinical microbiology practice. This will include sequence-based resistance testing and additional detection of particular virulence factors, making culture unnecessary on the intermediate or long term [184]. The generation of microbial sequence data for “short term” patient management will revolutionize infectiology and diagnostic microbiology, allowing for deeper and more rapid insights into the patients' infectious pathologies [90]. As a high-resolution tool, high-throughput sequencing has the potential to optimize both diagnostics and patient care [185]. NGS will affect antibiotic stewardship [80] by defining resistance by the presence of a mechanism rather than just in pharmacodynamic terms as it is performed right now. Present obstacles include the imperfect correlation of genotype and phenotype; further, technical challenges have to be overcome [80]. However, as NGS becomes increasingly cost effective and convenient, it bears the potential to replace the so far multiple and complex procedures in a microbiological routine laboratory by just a single, straightforward, and most efficient workflow [184].

Besides NGS, mass spectrometry will be the second key technique in rapid medical microbiology. The integration of subtype specific mass spectra databases in MS associated software packages will enable the identification of high-virulent strains within very short time periods. The mass spectrometric  *β*-lactamase assay (MSBL) as well as adaptations to other anti-microbiota classes will expectantly advance to helpful tools of the diagnostic microbiologist. Finally, the combination of both nucleic acid amplification and mass spectrometric analysis, for example, in PCR/ESI MS assays with its high multiplexing capacity, has the potential to enter routine diagnostic in the coming years.

Nevertheless, these highly sophisticated and expensive diagnostic solutions will hardly be available in resource-limited countries, for example, in the sub-Saharan tropics, where multidrug resistance is nevertheless on the rise [186]. Cheap and easy-to-perform rapid molecular techniques like fluorescence* in situ* hybridization (FISH) might be an option for such settings [187] until MALDI-TOF MS or sequence-based approaches become more affordable and easy to apply. The rapid and correct choice of adequate antibiotic therapy will decide on the survival of critically ill patients with infectious diseases, for example, sepsis patients [188, 189]. In times of decreasing susceptibility to antimicrobial drugs, this choice gets increasingly complicated. So the words of the ancient German infectious disease specialist Robert Koch become more and more true: “If a doctor walks behind his/her patient's coffin, sometime cause follows consequence.” (Original German text of the witticism: “Wenn ein Arzt hinter dem Sarg seines Patienten geht, so folgt manchmal die Ursache der Wirkung.”) Reliable information on the resistance patterns of etiologically relevant pathogens has to be rapidly available to avoid this final consequence as frequently as possible.

## Figures and Tables

**Table 1:** DNA-FISH-probes detecting clarithromycin resistance in *H*. *pylori, *Rüssmann et al., 2001a [93].

Target	Probe	Probe sequence
Wild type	ClaWT	5′-CGG-GGT-CTT-TCC-GTC-TT-3′
Clarithromycin resistance mutation 1 (A2143G)	ClaR1	5′-CGG-GGT-CTT-CCC-GTC-TT-3′
Clarithromycin resistance mutation 2 (A2144G)	ClaR2	5′-CGG-GGT-CTC-TCC-GTC-TT-3′
Clarithromycin resistance mutation 3 (A2143C)	ClaR3	5′-CGG-GGT-CTT-GCC-GTC-TT-3′

**Table 2:** PNA-FISH-probes detecting clarithromycin resistance in *H. pylori,* Cerqueira et al., 2013 [102], shortened versions of the DNA-FISH-probes from Table 1.

Target	Probe	Probe sequence
Wild type	HpWT	5′-GGT-CTT-TCC-GTC-T-3′
Clarithromycin resistance mutation 1 (A2143G)	Hp2	5′-GTC-TTC-CCG-TCT-T-3′
Clarithromycin resistance mutation 2 (A2144G)	Hp1	5′-GTC-TCT-CCG-TCT-T-3′
Clarithromycin resistance mutation 3 (A2143C)	Hp3	5′-GTC-TTG-CCG-TCT-T-3′

**Table 3:** DNA-FISH-probes detecting clarithromycin resistance in thermotolerant *Campylobacter *spp., Haas et al., 2008 [103]. Of note, probe C wt 23S is identical with probe ClaWT, probe C res 23S 2059A>G with probe ClaR2 (Table 1).

Target	Probe	Probe sequence
Wild type	C wt 23S	5′-CGG-GGT-CTT-TCC-GTC-TT-3′
Clarithromycin resistance mutation (A2059G)	C res 23S 2059A>G	5′-CGG-GGT-CTC-TCC-GTC-TT-3′

**Table 4:** DNA-FISH-probes detecting linezolid resistance in enterococci. Locked nucleic acids (LNA) were used at the mismatch position (bold, underlined print) within in probes.

Target	Probe	Probe sequence
Wild type	LZD-WT	5′-CCC-AGC-T**C**G-CGT-GC-3′
Linezolid resistance mutation (G2567T)	LZD-res	5′-CCC-AGC-T**A**G-CGT-GC-3′

**Table 5:** Approximate turn-around-time, investment costs, reagent costs, and necessity of skilled personnel of different rapid diagnostic test procedures.

Rapid diagnostic procedure	Turn-around-time	Investment costs	Reagents costs (per sample)	Necessity of skilled personnel
Agglutination assays	<5 minutes	—	<1.00€	Low
Fluorescence *in situ* hybridization	1-2 hours	<15,000.00€	1.00–8.00€	Intermediate
Real-time PCR (including DNA preparation)	4–6 hours	35,000.00–60,000.00€	15.00–25.00€	Strongly depending on the test system
Loop-mediated isothermal amplification (LAMP) assays	<1 hour	2,000.00–4,000.00€	15.00–25.00€	Intermediate
Next generation sequencing (NGS)	2–5 days	350,000.00–750,000.00€	75.00–800.00€	Very high
Matrix-assisted laser desorption ionization time-of-flight mass spectrometry (MALDI-TOF-MS)	<5 minutes	75,000.00–300,000.00€	<1.00€	High
